# Staphylococcal SplA and SplB serine proteases target ubiquitin(-like) specific proteases

**DOI:** 10.1186/s13568-025-01841-5

**Published:** 2025-02-22

**Authors:** Felix L. Glinka, Ole Schmöker, Abhishek K. Singh, Leif Steil, Christian Hentschker, Uwe Völker, Dominique Böttcher, Michael Lammers, Clemens Cammann, Ulrike Seifert, Elke Krüger, Michael Naumann, Barbara M. Bröker, Uwe T. Bornscheuer

**Affiliations:** 1 Department of Biotechnology & Enzyme Catalysis, Institute of Biochemistry, University of Greifswald, Greifswald, Germany; 2 Department of Synthetic and Structural Biochemistry, Institute of Biochemistry, University of Greifswald, Greifswald, Germany; 3 Friedrich Loeffler-Institute for Medical Microbiology-Virology, University Medicine Greifswald, Greifswald, Germany; 4 Department of Functional Genomics, Interfaculty Institute for Genetics and Functional Genomics, University of Greifswald, Greifswald, Germany; 5 Institute of Medical Biochemistry and Molecular Biology, University Medicine Greifswald, Greifswald, Germany; 6 Institute of Immunology, University Medicine Greifswald, Greifswald, Germany; 7 Institute of Experimental Internal Medicine, Otto Von Guericke University, Magdeburg, Germany

**Keywords:** Deubiquitination, Protein degradation, Serine protease-like, *Staphylococcus aureus*, SplA, SplB

## Abstract

*Staphylococcus aureus* is a Gram-positive opportunistic pathogen that has colonized nearly 30% of the human population and can cause life-threatening infections. *S. aureus* exports a variety of virulence factors, such as a novel set of extracellular serine protease-like proteins (Spls). Spls are expressed by most clinical isolates of *S. aureus*, but their pathophysiological substrates and role during the infection are largely unknown. Here we characterized the substrate and cleavage specificity of recombinantly expressed SplA and SplB proteins. We identified a group of ubiquitin or ubiquitin-like modifying enzymes including deubiquitinating enzymes from human as well as from bacterial sources to be so far unknown SplA and SplB substrates. Distinct cleavage sites within these substrates for SplA (YLY^↓^T, FMY^↓^N) and SplB (VCD^↓^S) were identified by mass spectrometry and confirmed by site-directed mutagenesis of the target proteins. Since many cellular immune signaling pathways are tightly regulated by ubiquitination, the specific cleavage of ubiquitin modifying enzymes strongly suggests a specific role of Spls in manipulating immune signaling and in competing with other bacteria.

**Supplementary Information:**

The online version contains supplementary material available at 10.1186/s13568-025-01841-5.

## Introduction

*Staphylococcus aureus* is a Gram-positive opportunistic pathogen that is persistently colonizing ~ 30% of the human population without visible adverse effects. At the same time the bacterium is a major cause of community-acquired and nosocomial infections that can result in life-threatening conditions (Lowy 1998; Tam and Torres 2019). With the ability to persist intracellularly, *S. aureus* can evade recognition and elimination by the host's immune system or antibiotic therapy, making it a source of recurrent infections (Strobel et al. 2016; Löffler et al. 2014). Additionally, occurrence of multi-drug resistant strains makes targeted and successful antibiotic treatment increasingly difficult (Lowy 1998; Tong et al. 2015).

*S. aureus* exports a variety of virulence factors, among them numerous proteases (Tam and Torres 2019). In 1996, a novel set of extracellular serine proteases was discovered: the serine protease-like proteins (Spls). The *spl* operon is located in the νSaβ pathogenicity island and is composed of six genes encoding the proteases named SplA to SplF (Rieneck et al. 1997; Reed et al. 2001). A study of the *spl* operon composition in nasal *S. aureus* isolates found that 82% of 96 investigated strains carry at least one *spl* gene, with 18.8% of the isolates having all six *spl* genes (Dasari et al. 2022). The frequent occurrence of these genes across different strains indicates an important function of the Spls. The operon is regulated alongside other *S. aureus* virulence factors (Gimza et al. 2019; Jenul and Horswill 2019), suggesting its involvement in virulence. Reintroducing the *spl* operon into an *S. aureus* strain devoid of other extracellular proteases, dramatically increased its virulence in murine bloodstream infection (Gimza et al. 2021). Although the Spls were discovered nearly three decades ago, their pathophysiological substrates and biological function during infection remains largely unknown.

Ubiquitination is a posttranslational modification involved in various pivotal cellular processes. The most prominent function is the targeting of protein substrates for their degradation by the proteasome to maintain cellular protein homeostasis. By regulating cellular signaling pathways, ubiquitination is associated with both innate and adaptive immune responses (Hu and Sun 2016; Park et al. 2014). Ubiquitination is characterized by the transfer of ubiquitin to a target protein in a three-step enzymatic process, the first step being mediated by an E1 activating enzyme. The second step is the transfer of ubiquitin to an E2 ubiquitin-conjugating enzyme, and in the third step the ubiquitin is transferred to specific lysine residues of the target protein by forming a complex with a specific E3 ubiquitin ligase and its substrate. The formation of ubiquitin chains can be reversed by deubiquitinating enzymes (DUBs), which hydrolyze the isopeptide bonds between ubiquitin moieties from the distal end of a chain or by cleaving entire chains by breaking the bond between the proximal ubiquitin and the substrate (Reyes-Turcu et al. 2009; Grumati and Dikic 2018).

In addition to ubiquitin, ubiquitin-like proteins, such as the small ubiquitin-like modifier (SUMO) are involved in various cellular processes. Similar to ubiquitination, the SUMO-proteins are transferred to target proteins (sumoylation) and impact their stability, activity, and interactions in cellular signaling pathways. Sumoylation has been associated with the regulation of signal transduction, stress responses, protein stability, and the cell cycle (Enserink 2015; Eifler et al. 2018). Proteolytic enzymes with specificities towards ubiquitin-like (Ubl) modifiers are classified as Ubl-specific proteases (ULPs) and small ubiquitin-like modifier (SUMO) specific proteases (SENPs), both of which belong to the cysteine protease superfamily.

During bacterial infection ubiquitination plays a central role in bacterial recognition and targeting, and regulating the clearance of pathogens by autophagy flux (Grumati and Dikic 2018). Several intracellular bacteria such as *Salmonella* or *Shigella* have been described to secrete effector proteins modulating ubiquitination and sumoylation processes promoting their intracellular survival by preventing recognition and elimination through infected host cells (Herhaus and Dikic 2018; Maculins et al. 2016; Liu et al. 2022).

Spls are natively expressed with an N-terminal signal peptide which is cleaved off precisely during secretion. This distinct N-terminal processing is necessary for Spl activity as even a single amino acid overhang greatly reduces or completely abolishes Spl activity, although the exact mechanism behind this is still unknown (Stec-Niemczyk et al. 2009; Pustelny et al. 2014).

We here aimed at characterization of substrates and cleavage specificities using SplA and SplB (recombinantly produced in *E. coli*). In the assays, we identified multiple ubiquitin-modifying enzymes being targeted by SplA and SplB. Proteolytic cleavage sites were identified by mass spectrometry (MS) and confirmed by site-directed mutagenesis (SDM) of key residues within those targets. A potential deregulation of ubiquitination and sumoylation events by *S. aureus* proteases might impact their intracellular survival and reflects a potential mechanism to evade cellular host defense mechanisms.

## Materials and methods

### Bacterial strains and plasmids

*Escherichia coli* strains TOP10 and BL21(DE3) were used for genetic manipulations and protein expression, respectively. The *spl* genes of *S. aureus* strain USA300 with an N-terminal SUMO-tag and C-terminal Twin-Strep-tag were ordered in a pET-28a(+) vector (BioCat GmbH, Heidelberg, Germany). The propeptide sequences were retained, while the signal peptide sequences were excluded in the design of the synthetic genes. The bacterial DUB and ULP genes were ordered from BioCat in a pET-45b(+) vector. The sequences were codon-optimized for expression in *E. coli*. USP48 cDNA containing a N-terminal His_6_ sequence was cloned in the pRSETC (Invitrogen) expression vector. The SENP1 gene in a pOPINS vector was kindly provided by Kay Hofmann (University of Cologne, Institute for Genetics). Sequences of the constructs are provided in the Supplementary Information.

### Recombinant production of proteins

SENP1, bacterial DUBs and Spl constructs were produced in *E. coli* BL21(DE3). The cells were grown in TB autoinduction media (TB, 25 mM Na_2_HPO_4_, 25 mM KH_2_PO_4_, 50 mM NH_4_Cl, 5 mM Na_2_SO_4_, 2 mM MgSO_4_, 0.5% (w/v) glycerol, 0.2% (w/v) lactose, 0.05% (w/v) glucose) at 37 °C until an OD_600_ of 0.8 was reached, at which point the temperature was reduced to 20 °C overnight. For SUMO-tag removal, the cell cultures of Spls and SENP1 were combined in a 2:1 ratio (v/v), centrifuged, resuspended in 100 mM TRIS (pH 8) and 150 mM NaCl, lysed by sonication and the lysate incubated for 1 h at 4 °C. USP48 was expressed in *E. coli* BL21(DE3)pLysS. The cells were transformed with the plasmid and incubated overnight at 28 °C. The overnight culture was diluted 1:200 and grown to OD_600_ of 0.7–0.8. Addition of 0.4 mM isopropyl 3-D-thiogalactopyranoside (IPTG) was followed by a 3 h incubation. The bacterial pellet was suspended in PBS buffer, disrupted by sonication and centrifuged. Commercially available recombinant proteases were received as follows: viral Sars-COV-2 Papain-like protease (Boston Biochem #E-611), USP7 (Boston Biochem #UC-355), OTUD7B (Boston Biochem #E-562) and UCHL3 (Antikoerper-online, Aachen, Germany, ABIN1638533).

### Protein purification

Recombinant Spls, SENP1 and target proteins were recovered from the lysate by gravity flow column protein purification. For Spl proteins, Strep-Tactin XT resin (IBA Lifesciences) was used. The column was equilibrated and washed with 100 mM TRIS buffer, 150 mM NaCl, pH 8. The elution buffer contained additional 50 mM biotin. The other proteins were purified by immobilized metal affinity chromatography (IMAC) using ROTI®Garose-His/Ni Beads (Roth). The column was equilibrated with 20 mM TRIS buffer, 300 mM NaCl, 10 mM imidazole, pH 7 and washed with 20 mM TRIS buffer, 500 mM NaCl, 20 mM imidazole, pH 7. The protein was eluted using 20 mM TRIS buffer, 300 mM NaCl, 250 mM imidazole, pH 7. Eluted proteins were desalted and the buffer exchanged to PBS with PD-10 columns (Sephadex G-25 resin, VWR). Lysate containing overexpressed USP48 was applied onto Ni^2+^-NTA resin columns (Qiagen). After excessive washing with PBS the bound protein was eluted with PBS containing 150 mM imidazole. The eluate was dialyzed against a low salt buffer. The purity of the proteins was validated by SDS-PAGE analysis.

### Proteolytic activity assays

If not stated otherwise, the target proteins were incubated with Spls in a 2:1 molar ratio at a concentration of 1 mg/mL in PBS at 37 °C for 24 h. The reaction was terminated by adding denaturing SDS sample buffer. The samples were then analyzed by SDS-PAGE or MS.

### Identification of fragments from Coomassie-stained SDS-PAGE gel slices

The Coomassie-stained gel slices were subjected to repeated washing with 200 µL ammonium bicarbonate buffer in 50% acetonitrile (ACN) for 15 min at 37 °C in a thermomixer shaker at 500 rpm until the staining was removed. Subsequently, the gel slices were dried by adding 15 µL of 100% ACN. After removal of the ACN, the dried gel slices were incubated in 10 µL of trypsin solution (10 ng/µL) at 37 °C overnight. If necessary, additional 20 mM ammonium bicarbonate buffer was added after one hour of incubation. The resulting peptides were extracted by incubation in an ultrasonic bath for 30 min in 20 µL of 0.1% acetic acid, followed by an additional 30 min incubation in 50% ACN in 0.05% acetic acid. Both extractions were collected by centrifugation and combined in a new vial. The extracted peptides were lyophilized and subsequently reconstituted in 12 µL 0.1% acetic acid prior to MS measurement.

### Identification of N-termini using the HUNTER approach

The identification of N-termini generated by SplA or SplB was conducted using a slightly modified HUNTER protocol as previously described by Weng et al. (2019). In brief, the proteins of interest were incubated with either SplA or SplB. Subsequently, the protein mixture and the corresponding control were subjected to dimethylation using either light or heavy formaldehyde. The dimethylated samples and corresponding controls were combined, bound to Sp3 beads, and digested using either trypsin, chymotrypsin, or GluC, according to the manufacturer's specifications. The resulting N-termini were coupled to undecanal and removed using C18 columns. The flow-through was subsequently lyophilized and reconstituted in 12 µL buffer A prior to MS measurement.

### MS measurement

LC-ESI–MS/MS analyses of the obtained peptide solutions were conducted using a reverse phase HPLC chromatography system (Ultimate 3000 nano-LC system, Thermo Fisher Scientific). This was either coupled to a Q Exactive Plus or an Exploris 480 mass spectrometer (Thermo Fisher Scientific), in accordance with the settings outlined in Tables S2 and S3.

Database searches were performed using Mascot (Matrix Science Inc.) for the gel slices and MaxQuant (Cox and Mann 2008) for the HUNTER analyses. Databases contained the proteins of interest and the proteins of the production strains (*E. coli* K12). Semi N-terminal cleavage rules for the used enzymes, two missed cleavages, variable modifications (methionine oxidation, N-terminal acetylation) and for the HUNTER samples the dimethyl labelling, N-terminal and at lysine, were considered. Further analyses were carried out in Scaffold 5 (Proteome Software Inc.), MS-Excel (Microsoft Corporation) and R. We used R version 4.4.1 (R Core Team 2024) and the following R packages: devtools v. 2.4.5 (Wickham et al. 2011), ggrepel v. 0.9.5 (Slowikowski 2016), ggsci v. 3.2.0 (Xiao 2016), htmlwidgets v. 1.6.4 (Vaidyanathan et al. 2014), openxlsx v. 4.2.6.1 (Schauberger and Walker 2014), plotly v. 4.10.4 (Sievert 2020), tidyverse v. 2.0.0 (Wickham et al. 2019).

## Results

### SENP1 cleavage by Spls

**Fig. 1:**
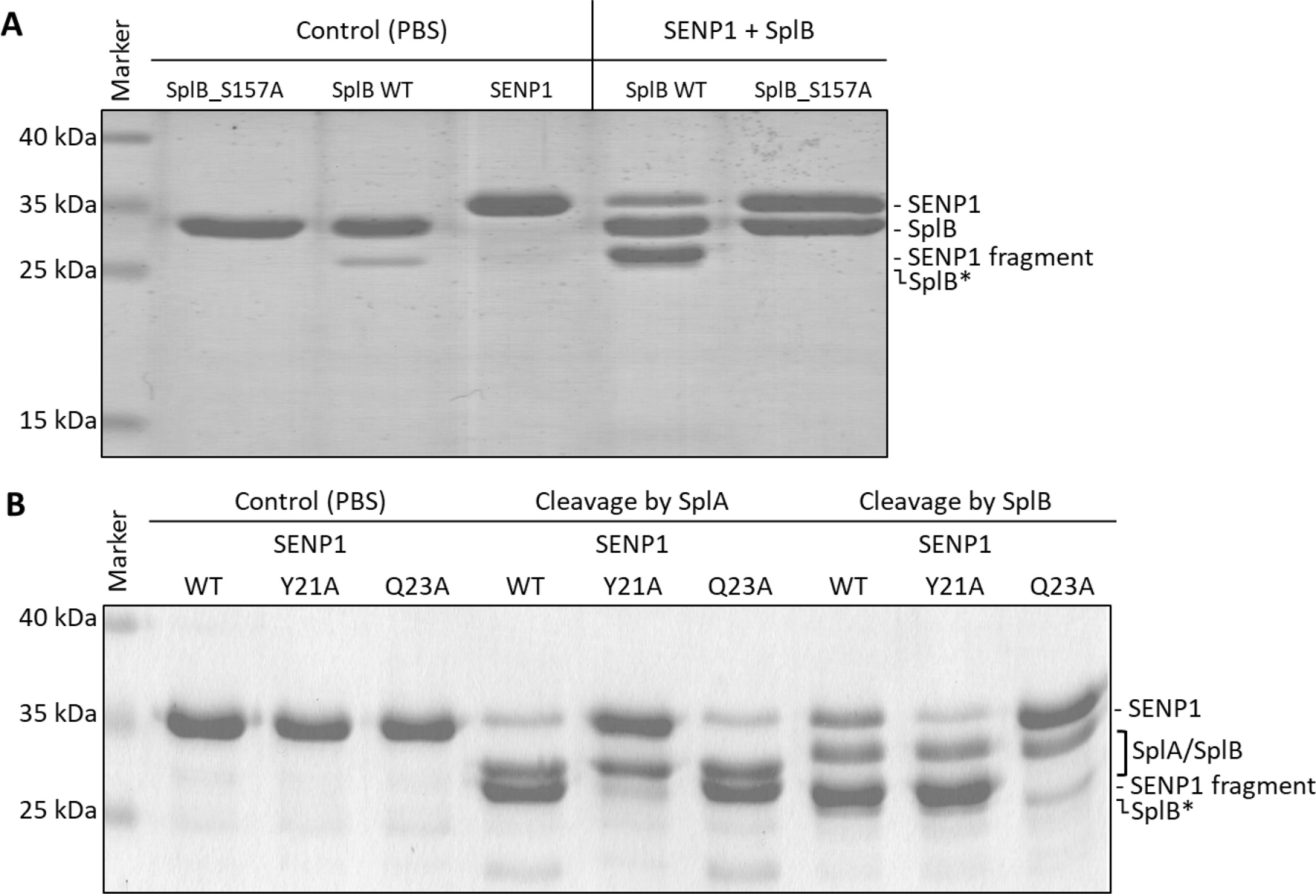
SDS-PAGE analysis of SENP1 cleavage by SplA or SplB. **A** SENP1 was incubated with the active SplB WT or the inactive SplB mutant S157A (with the catalytic serine exchanged to alanine) in a ~ 1:1 ratio for 3 h at 30 °C in PBS buffer. Control: incubation of Spl WT/S157A or SENP1 in PBS under same conditions. When incubated with SplB WT, a depletion of the SENP1 protein band (~ 35 kDa) was observed with a new band appearing at ~ 28 kDa. This was not the case for the inactive SplB mutant, indicating SENP1 cleavage catalyzed by SplB WT. **B** Cleavage site identification in SENP1. SplA and SplB predominantly cleaved at the TEV protease-cleavage motif of SENP1 at positions Y21 and Q23, respectively. SENP1 variants were incubated with Spls (2:1 ratio of SENP1 to Spls) for 24 h at 37 °C in PBS buffer. Control: incubation of SENP1 WT/Y21A or Q23A in PBS under same conditions. SplB* = SplB without C-terminal Twin-Strep-tag due to autohydrolysis (see Supplementary Information and Fig. S1 for further information)

The Spl constructs were recombinantly produced in *E. coli* with an N-terminal SUMO-tag that was then removed scarlessly by the human SUMO-protease SENP1, generating the native N-terminus and thereby activating the Spls. During SUMO-cleavage optimization, we observed a depletion of the SENP1 from the reaction mixture during the incubation. This was apparent due to the protein band of SENP1 (~ 35 kDa) missing in SDS-PAGEs following incubation with SplB in spite of the huge excess of SENP1 used for the SUMO-tag removal (~ 1:1 ratio of SENP1 to sumoylated SplB). Instead, a new band appeared at ~ 28 kDa. When SENP1 was incubated with the native SplB WT (purified after SUMO-tag removal), the same mass-shift of the SENP1 protein band of ~ 7 kDa was observed (Fig. 1A). This was not the case when SENP1 was incubated with the inactive SplB mutant S157A (with the catalytic serine exchanged to alanine). Further analysis revealed that the native SplA and SplB both predominantly cleaved the SENP1 construct at an N-terminal tobacco etch virus (TEV) protease-cleavage motif designed for tag removal. The truncation of ~ 20 AA (calculated mass: 3 kDa) resulted in the observed mass-shift of the SENP1 protein band of around 7 kDa (Fig. 1B). The Spl cleavage sites within the TEV protease-cleavage motif (SplA: ENLY^↓^FQG, SplB: ENLYFQ^↓^G, ^↓^ = cleavage site) were identified by MS and confirmed with SDM by exchanging the specific residues with alanine (Fig. 1B). Although the cleavage was subsequently determined to be inside the TEV protease-cleavage motif of the SENP1 construct instead of the SENP1 protein, the initially presumed cleavage of SENP1 lead to a screening for further target proteins similar to SENP1.

### Target screening for SplA and SplB

**Fig. 2:**
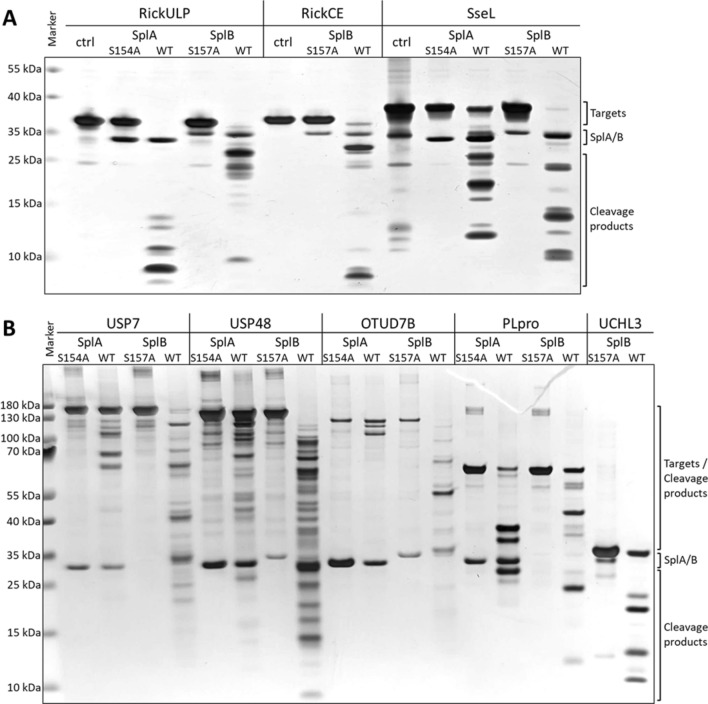
DUBs and ULPs targeted by SplA and SplB. Silver-stained SDS-PAGE of recombinantly expressed and purified Spl targets after incubation in PBS (ctrl), with inactive Spl mutants (SplA_S154A, SplB_S157A) and native Spls (WT) for 24 h at 37 °C. **A** Cleavage patterns of bacterial DUBs and ULPs catalyzed by Spls. **B** Cleavage patterns of human/viral DUBs and ULPs catalyzed by Spls

Based on the cleavage of SENP1 catalyzed by SplB, an extended screening for further target proteins, using both SplA and SplB, was performed using 13 DUBs and ULPs closely related to SENP1 that were available in our laboratory (Table 1). Most, but not all of these proteins were also cleaved by the Spls, indicating that these proteins also contain specific Spl cleavage sites. Inactive Spl mutants (mutation of catalytic residues: SplA_S154A, SplB_S157A) did not cleave any of the studied substrate candidates. The cleavage patterns of the identified Spl targets are shown in Fig. 2.

**Table 1:** Proteins used for SplA and SplB target screening

Protein/gene	Host organism	Protein function	Cleaved by	
			SplA	SplB
OTUD7B	*Homo sapiens*	DUB	Yes	Yes
SENP1		ULP (SUMO)	Yes*	Yes*
UCHL3		DUB	No	Yes
USP48		DUB	Yes	Yes
USP7		DUB	Yes	Yes
ElaD	*Escherichia coli*	DUB	No	No
Lpg1148	*Legionella pneumophila*	DUB	Yes	Yes
Lpg1621/ Ceg23		DUB	No	No
RavD		DUB	No	No
RickCE	*Rickettsia bellii*	DUB	Yes	Yes
RickULP		ULP	Yes	Yes
SnCE1	*Simkania negevensis*	DUB, ULP (SUMO)	No	No
SseL	*Salmonella typhimurium*	DUB	Yes	Yes
PLpro	*Severe acute respiratory syndrome coronavirus*	DUB, deISGylating	Yes	Yes

*Cleavage could only be confirmed inside an N-terminal TEV protease-cleavage motif of the SENP1 construct and not the SENP1 protein

### Cleavage site identification in the target proteins of Spls

Based on the results shown in Fig. 2, the cleavage sites in all these newly discovered target proteins needed to be identified. MS analysis of either single cleavage fragments (after gel extraction) or the whole reaction mixture with N-terminal labelling using the HUNTER method (Weng et al. 2019) was used to identify putative cleavage sites and thereby greatly reduce the amount of potential cleavage sites to be investigated by SDM. Table 2 lists the putative and confirmed cleavage sites for four candidate proteins, which could be determined using these methods.

**Table 2:** Cleavage sites identified by MS-analysis

Target protein	Cleavage sites (P1 residues)	
	SplA	SplB
RickULP	E35, **Y84**, F102, **Y162**, Y166, Y179	H106, H107, D209*
RickCE	N79, R108, R117, Q124, M151	Q30, D32, Q72, E73, N79, R97, Q123, N227, A232
SseL	F52, F54, Y278, F299	Q80, Q130, D132, Q133, S285, Q289, F299, Q311, R319
SENP1	**Y21**, F52, R53, N54, R235, F239, R250	**Q23**, R53, N54, Q57, D167, R235

Cleavage site candidates (P1 residues, nomenclature according to Schechter and Berger (1967) for SplA and SplB were determined by gel-extracted single cleavage fragments or using the HUNTER method. Cleavage sites confirmed by site-directed mutagenesis (exchange to alanine) are marked bold

*See Fig. S2

**Fig. 3:**
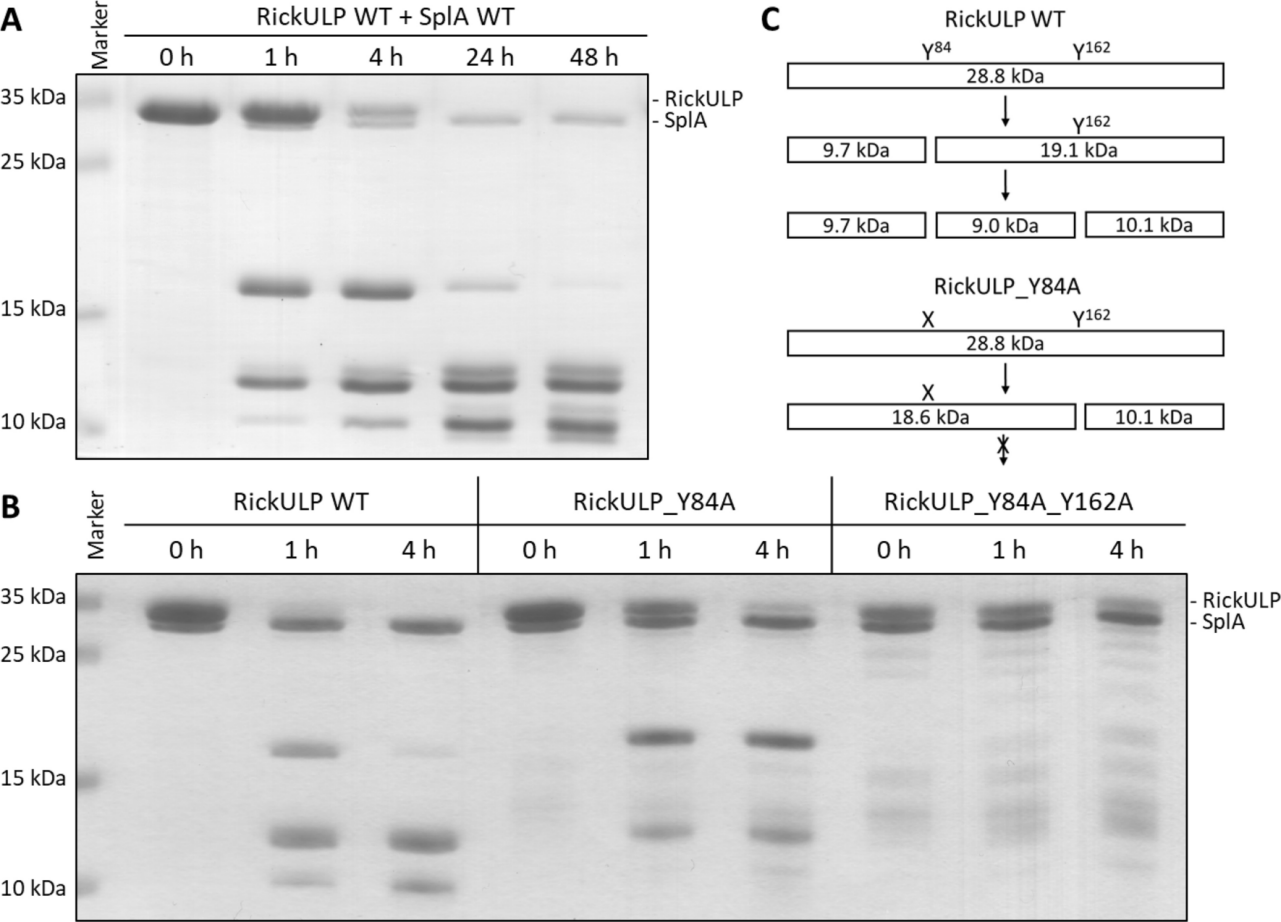
SplA cleavage sites in RickULP. **A** SDS-PAGE analysis of RickULP WT cleavage catalyzed by SplA over time in PBS at 37 °C (5:1 ratio of RickULP to SplA). **B** SDS-PAGE of RickULP WT, Y84A mutant and Y84A_Y162A double mutant cleavage catalyzed by SplA in PBS at 37 °C (2:1 ratio of RickULP to SplA). **C** Scheme of RickULP WT and Y84A mutant cleavage by SplA at the two determined cleavage sites Y84 and Y162 including the calculated mass of the cleavage products. X = residue Y84 exchanged to alanine.

Most of the newly identified targets were cleaved slowly, requiring an excess of Spls and prolonged incubation times. Since the cleavage of RickULP by SplA is comparatively fast and generates clear distinct bands in SDS-PAGEs (Fig. 3A), it was selected as an example to validate the determined cleavage sites by SDM. For RickULP, two prime cleavage sites were identified at residues Y84 (YLY^↓^T) and Y162 (FMY^↓^N). The cleavage site Y84 appears to be preferred by SplA, resulting in ~ 17 kDa (calculated mass: 19.1 kDa) and ~ 12 kDa (calculated mass: 9.7 kDa) fragments. The larger fragment is then further digested at the cleavage site Y162, resulting in ~ 12.5 kDa (calculated mass: 10.1 kDa) and ~ 10 kDa (calculated mass: 9 kDa) fragments. Exchanging Y84 with alanine by SDM resulted in Y162 as the primary cleavage site, generating fragments of similar mass as the Y84 cleavage site (calculated mass of 18.9 and 10.1 kDa), but no further digestion of both fragments was observed afterwards. After exchanging both Y84 and Y162 with alanine, none of the previously clearly visible cleavage products were observed (Fig. 3B). This clearly confirms that these two positions are prime cleavage sites within RickULP for SplA as identified by MS.

## Discussion

In this study we identified several new targets of the *S. aureus* serine protease-like proteins SplA and SplB, determined the cleavage sites with MS and confirmed several of them with SDM by introduction of alanine mutations. All newly identified targets are ubiquitin(-like)-specific proteases, indicating a specificity of Spls towards this group of proteins.

MS analysis was used to identify putative cleavage sites that were then investigated by SDM. Due to the enrichment used in the HUNTER method, we likely see some artifacts among the identified putative cleavage sites that we cannot see in the SDS-PAGE. This was also observed in the data published by Weng et al. (2019), especially for arginine residues.

The native SplA and SplB constructs cleaved the SENP1 construct predominantly at an N-terminal TEV protease-cleavage motif designed for tag removal (SplA: ENLY^↓^FQG, SplB: ENLYFQ^↓^G, ^↓^ = cleavage site). The TEV protease-cleavage motif being targeted was previously described for SplB (Knyphausen et al. 2023), but not yet known for SplA.

SplA and SplB cleavage motifs were previously determined by CLiPS (cellular library of peptide substrates) and PS-SCL (positional scanning substrate libraries). A search of the human proteome with the determined consensus cleavage sequences (SplA: YLYS, SplB: WELQ) was used to identify putative Spl substrates but did not identify deubiquitinating enzymes as putative targets (Stec-Niemczyk et al. 2009; Dubin et al. 2008). In addition, the putative targets of SplA include three E3 ubiquitin ligases: ubiquitin conjugation factor E4 A, E3 ubiquitin-protein ligase TRAF7 and NEDD4-like E3 ubiquitin-protein ligase (Stec-Niemczyk et al. 2009).

Out of all newly identified targets, only RickULP contains the entire SplA consensus cleavage sequence (YLYT). Otherwise, only partial consensus cleavage sequences of SplA and SplB are present. However, the previously determined cleavage motifs of SplA and SplB also showed some promiscuity in the CLiPS or PS-SCL analyses and none of the previously identified cleavage sites in actual proteins contained the entire consensus cleavage sequences (Dasari et al. 2022; Stec-Niemczyk et al. 2009; Dubin et al. 2008).

SplA-F share 43.9 to 94.6% sequence identity to each other and, as a group, contain significant sequence similarity to the *S. aureus* serine protease V8 (33–36%) and exfoliative toxins (Reed et al. 2001). V8 protease is known to contribute to immune evasion (Tam and Torres 2019). Additionally, a recent study also showed that SplB contributes to *S. aureus* immune evasion by inactivating central human complement proteins (Dasari et al. 2022).

The previously unknown targets identified here include human DUBs that are known to be involved in immune signaling pathways (Xiang et al. 2020), including inflammation and T cell activation (Hu et al. 2016). USP7 stabilizes MYD88, an important factor of the Toll-like receptor (TLR)-mediated activation of the nuclear factor “kappa light-chain enhancer” of activated B cells (NF-κB) pathway (Zhang et al. 2022). Additionally, USP7 is connected to the activation of the inflammasome (Palazón-Riquelme et al. 2018) and attenuates endoplasmic reticulum stress-induced cell death (Lee and Chung 2023). USP48 is implicated in activation of NF-κB (Mirra et al. 2022; Schweitzer and Naumann 2015; Ghanem et al. 2019), induces pyroptosis by stabilization of Gasdermin E (Ren et al. 2023) and promotes cell survival in *Helicobacter pylori* infection (Jantaree et al. 2022). OTUD7B (Cezanne) prevents tumor necrosis factor (TNF)-induced apoptosis of dendric cells in infection (Harit et al. 2023) and facilitates T cell activation and inflammatory responses by regulating Zap70 ubiquitination (Hu et al. 2016). Furthermore, OTUD7B is involved in mTORC2 signaling (Wang et al. 2017). UCHL3 plays a critical role in tumorigenesis, cancer progression and the onset of neurological disorders (Lei et al. 2024). UCHL3 also enhances interferon alpha and beta receptor subunit 1 expression leading to increased IFN-I mediated signaling pathway and antiviral activity (Zhao et al. 2017). Thus, specific cleavage of these DUBs and subsequent disruption of their respective signaling pathways could therefore modify immune signaling to *S. aureus* infection.

The selection of proteins for the target screening for SplA and SplB performed in this work consisted of 14 closely related proteins belonging to the group of ubiquitin(-like)-specific proteases (Table 1). While sharing high sequence and structure similarity among each other, most but not all of the potential target proteins were cleaved by the Spls. This indicates Spl specificity towards certain (host) ubiquitin factors. This selection of targets only allowed for the discovery of targeted ubiquitin factors and did not ascertain if other non-ubiquitin factors are targeted as well. Therefore, it is possible that the specificity of Spl proteases is not exclusively towards ubiquitin factors.

The results obtained so far reflect an in vitro connection between Spls and the investigated target proteins, whether this cleavage has in vivo relevance needs to be investigated in further experiments. First data in a proteome analysis from *S. aureus* infected macrophage-like cell line THP1 showed a decrease in the abundance of the analyzed proteins USP7, USP48, UCHL3, OTUD7B, and SENP1 (data not shown) which might indicate an intracellular role of Spls during infection.

To date, bacterial proteases that specifically target the ubiquitin system by specific proteolytic cleavages have only been described for DUBs from Gram-negative bacteria (Hermanns and Hofmann 2019; Schubert et al. 2020). To the best of our knowledge, Spls of *S. aureus* are therefore the first known example of proteases in Gram-positive bacteria that specifically can cleave ubiquitin factors of the host cell or that of competing bacteria. For Gram-positive bacteria, only indirect interference on the ubiquitin system is known, such as surface proteins recruiting host proteins to prevent ubiquitination (Yoshikawa et al. 2009) or a secreted protease cleaving autophagy adaptor proteins that recognize ubiquitinated proteins (Barnett et al. 2013).

Thus, these findings may present an exciting new avenue for future research to help understanding the role of Spls during *S. aureus* infection.

## Supplementary Information

Additional file1 (PDF 955 kb)

## Data Availability

The datasets generated and/or analyzed during the current study are available from the corresponding author on reasonable request. MassIVE (MSV000096077), 10.25345/C5K931J4J.

## Abbreviations

DUB: Deubiquitinating enzyme
IMAC: Immobilized metal affinity chromatography
IPTG: 3-D-thiogalactopyranoside
MS: Mass spectrometry
SDM: Site-directed mutagenesis
SDS-PAGE: Sodium dodecyl sulfate–polyacrylamide gel electrophoresis
SENP: Small ubiquitin-like modifier specific protease
Spls: Serine protease-like proteins
SUMO: Small ubiquitin-like modifier
TEV: Tobacco etch virus
Ubl: Ubiquitin-like
ULP: Ubl-specific protease
