# Supplementary material for: Staphylococcal SplA and SplB serine proteases target ubiquitin(-like) specific proteases
Source: AMB Express. 2025 Feb 22;15:32. doi: 10.1186/s13568-025-01841-5 (PMC11846797; doi:10.1186/s13568-025-01841-5)
Supplement: Supplementary file 1 — Additional file1 (PDF 955 kb) [file 13568_2025_1841_MOESM1_ESM.pdf]

Supplementary Information

Staphylococcal SplA and SplB serine proteases target ubiquitin(-like) specific proteases

Felix L. Glinka[1], Ole Schmöker[2], Abhishek K. Singh[3], Leif Steil[4], Christian Hentschker[4], Uwe Völker[4], Dominique Böttcher[1], Michael Lammers[2], Clemens Cammann[3], Ulrike Seifert[3], Elke Krüger[5], Michael Naumann[7], Barbara M. Bröker[6], Uwe T. Bornscheuer[1*]

SplB autohydrolysis of C-terminal Twin-Strep-tag

The native SplB construct used in this study shows an additional ~28 kDa band in SDS-PAGEs, which is not present in preparations of the inactive SplB mutant or SplA (Figure S1B). This ~28 kDa band increases in intensity with incubation times (e.g. during cleavage assays). MS-analysis of the lower band revealed a C-terminal truncation of the SplB construct, specifically inside the Twin-Strep-tag. The cleavage was confirmed by MS to occur after the glutamine residues in the first and second Strep-repeat (WSHPQ↓FEK, ↓ = cleavage site). Since glutamine was previously determined to be accepted in the P1 position (nomenclature according to Schechter and Berger (Schechter and Berger 1967)) of the SplB cleavage motif (Dubin et al. 2008), this is likely due to the recombinant SplB construct targeting its own C-terminal Twin-Strep-tag leading to autohydrolysis.

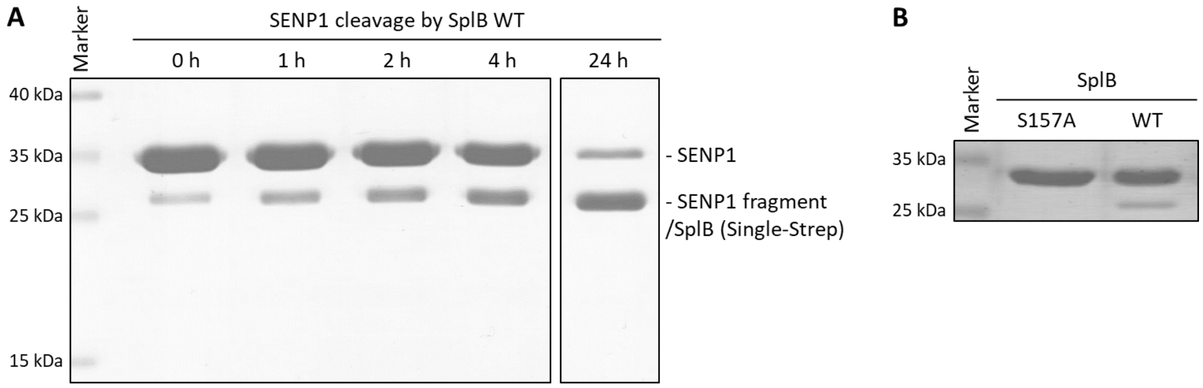

Fig. S1 SENP1 cleavage by SplB and SplB autohydrolysis. **A:** Time-dependent cleavage of SENP1 by SplB (construct with Single-Strep-tag). SENP1 was incubated with SplB (~10:1 ratio of SENP1 to SplB) at 37 °C in PBS buffer. The depletion of the SENP1 protein band (~35 kDa) and simultaneous increase of the lower band (~28 kDa, SplB and SENP1 fragment signals overlaid) signifies the time-dependent cleavage of SENP1 catalyzed by SplB. **B:** The recombinant SplB construct cleaved-off its own Twin-Strep-tag after the Gln residues present in both Strep repeats (WSHPQ↓FEK, ↓ = cleavage site). The enzymatically inactive SplB construct was unable to cleave its Twin-Strep-tag. Both protein samples shown were taken directly after purification. SplB_S157A = inactive SplB mutant; WT = native SplB. Additional band appearing for SplB WT at ~28 kDa.

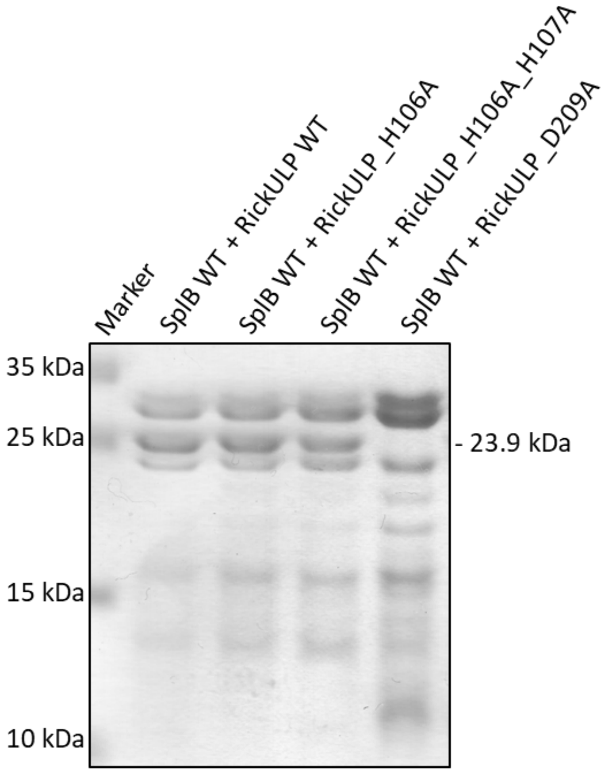

Fig. S2 Cleavage patterns of RickULP variants digested by SplB. Confirmation of SplB cleavage sites in RickULP identified using the HUNTER method by site-directed mutagenesis. SDS-PAGE of recombinantly expressed and purified RickULP variants after incubation with native SplB (WT) for 24 h at 37 °C. Cleavage of RickULP at residue D209 would result in a 23.9 kDa fragment, which was not observed for the D209A mutant.

Table S1 Amino acid sequences of the protein constructs.

Construct	Amino acid sequence
ElaD	MVTVVSNYCQLSQTQQLSQTFAEKFTVTEELLQSLKKTALSGDEESIELLHNIALGYDKFGKEAEDILYHIVR<br>TPTNETLSIIRLIKNACLKLYNLAHIATNSPLKSHDSDDLLFKKLFSPSKLMTIIGDEIPLISEKQSLSKVLL<br>NDENNELSDGTNFWDKNRQLTTDEIACYLQKIAANAKNTQVNYPTGLYVPYSTRTHLEDALNENIKSDPSWPN<br>EVQLFPINTGGHWILVSLQKIVNKKNNKLQIKCVIFNSLRALGYDKENSLKRVINSFNSELMGEMSNNNIKVH<br>LNEPEIIFLHADLQQYLSQSCGAFVCMAAQEVIEQRESNSDSAPYTLLKNHADRFKKYSAEEQYEIDFQHRLA<br>NRNCYLDKYGDANINHYYRNLEIKHSQPKNRASGKRVS
Lpg1148 (1-305)	MYMDLGSDNMALSKEIQNKSLRSLIQKYARQLNETDKKLIELINNLAAADDIPLYAQNQNVINLLIKTIKDSQ<br>SEHNPDLEGTSYFIYGLITAEMEAQSEINRQRHLMQGRFDETALHLRTERDQMQDHDMTLLMPKSQGRIVVMA<br>VLNRYDSHSANAIIETLASDVFNPEVHYIMIPVGPGHWRGVYLSKPQGGTSDTAYDLELFDPYGPEGAAVLDD<br>YVLDLLNQCGVPKELVNIRHTGPKHPQGDAYSCGDFTCAYSHKKMKEFGAPEGSYNPILIDTLDNLGNEDNVL<br>RMTTREETRALVD
Lpg1621/ Ceg23 (1-368)	MHTKKDKKVISLQERVENAVDVSGAFDNCFFHNFALYLLTNNLPLPDDLFHFKSIINRNSKAEQLFEFFHNPE<br>SLNLFSILDKENDVSEPSGYLFEKSLILGFLLREWFPTQLVNNSAVKAEMLEGEKGVFSAFKNYKEYRSFMSK<br>EELKSTEFGALYEANEAFLEYFYNRSESTLINKDSPFEKYFVGSSSDEEAIKNYWDAEGYTLYCQHLAKPQVK<br>LSYIEIMTMMKVINQPLTIYDRSTSSIVAEYVNPKVNLPDFEVAIDALQGHYFLLKTEETEKELEEYERSYAQ<br>YKRDRSEILAHSDKPVSSLLVRATCPKGHLDEDPFIALIESLSEINSLSQIDTNLKNENTDIPNCNFLLKVGA<br>SVV
OTUD7B	MAHHHHHHTLDMDAVLSDFVRSTGAEPGLARDLLEGKNWDVNAALSDFEQLRQVHAGNLPPSFSEGSGGSRTP<br>EKGFSDREPTRPPRPILQRQDDIVQEKRLSRGISHASSSIVSLARSHVSSNGGGGGSNEHPLEMPICAFQLPD<br>LTVYNEDFRSFIERDLIEQSMLVALEQAGRLNWWVSVDPTSQRLLPLATTGDGNCLLHAASLGMWGFHDRDLM<br>LRKALYALMEKGVEKEALKRRWRWQQTQQNKESGLVYTEDEWQKEWNELIKLASSEPRMHLGTNGANCGGVES<br>SEEPVYESLEEFHVFVLAHVLRRPIVVVADTMLRDSGGEAFAPIPFGGIYLPLEVPASQCHRSPLVLAYDQAH<br>FSALVSMEQKENTKEQAVIPLTDSEYKLLPLHFAVDPGKGWEWGKDDSDNVRLASVILSLEVKLHLLHSYMNV<br>KWIPLSSDAQAPLAQPESPTASAGDEPRSTPESGDSDKESVGSSSTSNEGGRRKEKSKRDREKDKKRADSVAN<br>KLGSFGKTLGSKLKKNMGGLMHSKGSKPGGVGTGLGGSSGTETLEKKKKNSLKSWKGGKEEAAGDGPVSEKPP<br>AESVGNGGSKYSQEVMQSLSILRTAMQGEGKFIFVGTLKMGHRHQYQEEMIQRYLSDAEERFLAEQKQKEAER<br>KIMNGGIGGGPPPAKKPEPDAREEQPTGPPAESRAMAFSTGYPGDFTIPRPSGGGVHCQEPRRQLAGGPCVGG

	LPPYATFPRQCPPGRPYPHQDSIPSLEPGSHSKDGLHRGALLPPPYRVADSYSNGYREPPEPDGWAGGLRGLP<br>PTQTKCKQPNCSFYGHPETNNFCSCCYREELRRREREPDGELLVHRF
PLpro	MAHHHHHHGADVTKIKPHVNHEGKTFFVLPSDDTLRSEAFEYYHTLDESFLGRYMSALNHTKKWKFPQVGGLT<br>SIKWADNNCYLSSVLLALQQLEVKFNAPALQEAYYRARAGDAANFCALILAYSNKTVGELGDVRETMTHLLQH<br>ANLESAKRVLNVVCKHCGQKTTTLTGVEAVMYMGTLSYDNLKTGVSIPCVCGRDATQYLVQQESSFVMMSAPP<br>AEYKLQQGTFLCANEYTGNYQCGHYTHITAKETLYRIDGAHLTKMSEYKGPVTDVFYKETSYTTTIKPVSYKL<br>DGVTYTEIEPKLDGYYK
RavD	MNLKKEVFLNQNCAEMMIKKAARLILGSDLDFEYTRDVQDIQIDLGPAFMFSPDEEKTLWVSGKNQETLERDL<br>ATLNKSSAYFFRTGTQGGAGHWQVLYYEAAKSGWVSYSSQSNHFQVTDSNGKLTASGKGLLVPHANWGKENGN<br>YAFLLVNASAQNIIHAANFVYIFRTQNEDAAIQYCALNQALHPEIKRITITKVQTQTRLTPEIHCPRGDLSYA<br>NLKNVYSKHLALLHVINRLEAGSKSYNPYWMGSQEKLNKIFEALGHCYQKGLDVDNELNNSNSKLSKAINMRR<br>LPSLFSFWCEKNESFVESRKIIEEHRICRLNNL
RickCE	MAHHHHHHVGTEIKDYWYTENEITHLLTAQLDEKKFSVQPAITFRNTALTEEMLKDYTAKGEEKNKILAEVQE<br>TIKIANLIPDKEERALMLGDAKKREEILKLSDAEREKLKNDLLRGGEAQQQINEDILNRATKDIKDNGKEAAV<br>IPIEMGYGHWTVLVAKYDKKDNQIILTFNDSLGNSINYDGQKLPKLIDKTLGNLPNKPIIIDEQTKQQTDQSA<br>CGVFTVDNGIKIAKGQAILSTEESKGEKGLRLREHHAQILTDAMFKQDAQWIRQQ
RickULP	MAHHHHHHVGTQQQAPANNQKPWEKLGIPQEMYKESLKAEQQLAKPIIEPKQQIPEKKSSLVINTEDQVGVYN<br>TGNIKQPTYLYTEDDIKNILEANIDKNMFSIFHHASLEEPEILKDTLRVTVEDLILDNKPAIIPLNTGHKHWL<br>LLMASKDDKGNINFMYNDPYGEPLESQPKVTEYITEIYPDAKITDLNTKQQANVYDCGVFVCDSAIKLSKGQK<br>ILTTEESKDQGINLRQAQANTLLIQQQAITIGHE
SENP1	MSYYHHHHHHDYDIPTTENLYFQGAMADIGSDSEDEFPEITEEMEKEIKNVFRNGNQDEVLSEAFRLTITRKD<br>IQTLNHLNWLNDEIINFYMNMLMERSKEKGLPSVHAFNTFFFTKLKTAGYQAVKRWTKKVDVFSVDILLVPIH<br>LGVHWCLAVVDFRKKNITYYDSMGGINNEACRILLQYLKQESIDKKRKEFDTNGWQLFSKKSQEIPQQMNGSD<br>CGMFACKYADCITKDRPINFTQQHMPYFRKRMVWEILHRKLL
SnCE1	GPLGSSLTSKAICRYSLSSVESIQPKTQVKAIEPKGEKDRFLIDERNWLNNQHLGLYSLFLQEKYGPEVFFPF<br>GGWTYVFPGLTDRFFKEDSYHILDVRAKRIKSFLDYKSITYPLFIGGNHWGLLFIDREKRTVEYYDSKINYGN<br>YEEGLQGIKDVAAKFTKYDPGEKPYTYLEKIKKKLQPDGYQCGPWALYFLEHRLENPEVDFNQLDLNEAQNMI<br>AKYRFAVRDKLLELQKNGNTLYC
SplA WT	EKNVKEITDATKEPYNSVVAFVGGTGVVVGKNTIVTNKHIAKSNDIFKNRVSAHHSSKGKGGGNYDVKDIVEY<br>PGKEDLAIVHVHETSTEGLNFNKNVSYTKFADGAKVKDRISVIGYPKGAQTKYKMFESTGTINHISGTFMEFD<br>AYAQPGNSGSPVLNSKHELIGILYAGSGKDESEKNFGVYFTPQLKEFIQNNIEKGSWSHPQFEKGGGSGGGSG<br>GSSAWSHPQFEK
SplA-SUMO	MGHHHHHHGSGLVPRGSASMSDSEVNQEAKPEVKPEVKPETHINLKVSDGSSEIFFKIKKTTPLRRLMEAFAK<br>RQGKEMDSLRFLYDGIRIQADQTPEDLDMEDNDIIEAHREQIGGEKNVKEITDATKEPYNSVVAFVGGTGVVV<br>GKNTIVTNKHIAKSNDIFKNRVSAHHSSKGKGGGNYDVKDIVEYPGKEDLAIVHVHETSTEGLNFNKNVSYTK<br>FADGAKVKDRISVIGYPKGAQTKYKMFESTGTINHISGTFMEFDAYAQPGNSGSPVLNSKHELIGILYAGSGK<br>DESEKNFGVYFTPQLKEFIQNNIEKGSWSHPQFEKGGGSGGGSGGSSAWSHPQFEK
SplB WT	ENNVTKVKDTNIFPYTGVVAFKSATGFVVGKNTILTNKHVSKNYKVGDRITAHPNSDKGNGGIYSIKKIINYP<br>GKEDVSVIQVEERAIERGPKGFNFNDNVTPFKYAAGAKAGERIKVIGYPHPYKNKYVLYESTGPVMSVEGSSI<br>VYSAHTESGNSGSPVLNSNNELVGIHFASDVKNDDNRNAYGVYFTPEIKKFIAENIDKGSWSHPQFEKGGGSG<br>GGSGGSSAWSHPQFEK
SplB-SUMO	MGHHHHHHGSGLVPRGSASMSDSEVNQEAKPEVKPEVKPETHINLKVSDGSSEIFFKIKKTTPLRRLMEAFAK<br>RQGKEMDSLRFLYDGIRIQADQTPEDLDMEDNDIIEAHREQIGGENNVTKVKDTNIFPYTGVVAFKSATGFVV<br>GKNTILTNKHVSKNYKVGDRITAHPNSDKGNGGIYSIKKIINYPGKEDVSVIQVEERAIERGPKGFNFNDNVT<br>PFKYAAGAKAGERIKVIGYPHPYKNKYVLYESTGPVMSVEGSSIVYSAHTESGNSGSPVLNSNNELVGIHFAS<br>DVKNDDNRNAYGVYFTPEIKKFIAENIDKGSWSHPQFEKGGGSGGGSGGSSAWSHPQFEK
SseL	MAHHHHHHVGTMSDEALTLLFSAVENGDQNCIDLLCNLALRNDDLGHRVEKFLFDLFSGKRTGSSDIDKKINQ<br>ACLVLHQIANNDITKDNTEWKKLHAPSRLLYMAGSATTDLSKKIGIAHKIMGDQFAQTDQEQVGVENLWCGAR<br>MLSSDELAAATQGLVQESPLLSVNYPIGLIHPTTKENILSTQLLEKIAQSGLSHNEVFLVNTGDHWLLCLFYK<br>LAEKIKCLIFNTYYDLNENTKQEIIEAAKIAGISESDEVNFIEMNLQNNVPNGCGLFCYHTIQLLSNAGQNDP<br>ATTLREFAENFLTLSVEEQALFNTQTRRQIYEYSLQ
UCHL3	MAHHHHHHEGQRWLPLEANPEVTNQFLKQLGLHPNWQFVDVYGMDPELLSMVPRPVCAVLLLFPITEKYEVFR<br>TEEEEKIKSQGQDVTSSVYFMKQTISNACGTIGLIHAIANNKDKMHFESGSTLKKFLEESVSMSPEERARYLE<br>NYDAIRVTHETSAHEGQTEAPSIDEKVDLHFIALVHVDGHLYELDGRKPFPINHGETSDETLLEDAIEVCKKF<br>MERDPDELRFNAIALSAA
USP48	MAHHHHHHAPRLQLEKAAWRWAETVRPEEVSQEHIETAYRIWLEPCIRGVCRRNCKGNPNCLVGIGEHIWLGE<br>IDENSFHNIDDPNCERRKKNSFVGLTNLGATCYVNTFLQVWFLNLELRQALYLCPSTCSDYMLGDGIQEEKDY<br>EPQTICEHLQYLFALLQNSNRRYIDPSGFVKALGLDTGQQQDAQEFSKLFMSLLEDTLSKQKNPDVRNIVQQQ<br>FCGEYAYVTVCNQCGRESKLLSKFYELELNIQGHKQLTDCISEFLKEEKLEGDNRYFCENCQSKQNATRKIRL<br>LSLPCTLNLQLMRFVFDRQTGHKKKLNTYIGFSEILDMEPYVEHKGGSYVYELSAVLIHRGVSAYSGHYIAHV<br>KDPQSGEWYKFNDEDIEKMEGKKLQLGIEEDLAEPSKSQTRKPKCGKGTHCSRNAYMLVYRLQTQEKPNTTVQ<br>VPAFLQELVDRDNSKFEEWCIEMAEMRKQSVDKGKAKHEEVKELYQRLPAGAEPYEFVSLEWLQKWLDESTPT<br>KPIDNHACLCSHDKLHPDKISIMKRISEYAADIFYSRYGGGPRLTVKALCKECVVERCRILRLKNQLNEDYKT<br>VNNLLKAAVKGSDGFWVGKSSLRSWRQLALEQLDEQDGDAEQSNGKMNGSTLNKDESKEERKEEEELNFNEDI<br>LCPHGELCISENERRLVSKEAWSKLQQYFPKAPEFPSYKECCSQCKILEREGEENEALHKMIANEQKTSLPNL<br>FQDKNRPCLSNWPEDTDVLYIVSQFFVEEWRKFVRKPTRCSPVSSVGNSALLCPHGGLMFTFASMTKEDSKLI<br>ALIWPSEWQMIQKLFVVDHVIKITRIEVGDVNPSETQYISEPKLCPECREGLLCQQQRDLREYTQATIYVHKV

	VDNKKVMKDSAPELNVSSSETEEDKEEAKPDGEKDPDFNQSNGGTKRQKISHQNYIAYQKQVIRRSMRHRKVR<br>GEKALLVSANQTLKELKIQIMHAFSVAPFDQNLSIDGKILSDDCATLGTLGVIP
USP7	MAHHHHHHNHQQQQQQKAGEQQLSEPEDMEMEAGDTDDPPRITQNPVINGNVALSDGHNTAEEDMEDDTSWR<br>SEATFQFTVERFSRLSESVLSPPCFVRNLPWKIMVMPRFYPDRPHQKSVGFFLQCNAESDSTSWSCHAQAVLK<br>IINYRDDEKSFSRRISHLFFHKENDWGFSNFMAWSEVTDPEKGFIDDDKVTFEVFVQADAPHGVAWDSKKHTG<br>YVGLKNQGATCYMNSLLQTLFFTNQLRKAVYMMPTEGDDSSKSVPLALQRVFYELQHSDKPVGTKKLTKSFGW<br>ETLDSFMQHDVQELCRVLLDNVENKMKGTCVEGTIPKLFRGKMVSYIQCKEVDYRSDRREDYYDIQLSIKGKK<br>NIFESFVDYVAVEQLDGDNKYDAGEHGLQEAEKGVKFLTLPPVLHLQLMRFMYDPQTDQNIKINDRFEFPEQL<br>PLDEFLQKTDPKDPANYILHAVLVHSGDNHGGHYVVYLNPKGDGKWCKFDDDVVSRCTKEEAIEHNYGGHDDD<br>LSVRHCTNAYMLVYIRESKLSEVLQAVTDHDIPQQLVERLQEEKRIEAQKRKERQEAHLYMQVQIVAEDQFCG<br>HQGNDMYDEEKVKYTVFKVLKNSSLAEFVQSLSQTMGFPQDQIRLWPMQARSNGTKRPAMLDNEADGNKTMIE<br>LSDNENPWTIFLETVDPELAASGATLPKFDKDHDVMLFLKMYDPKTRSLNYCGHIYTPISCKIRDLLPVMCDR<br>AGFIQDTSLILYEEVKPNLTERIQDYDVSLDKALDELMDGDIIVFQKDDPENDNSELPTAKEYFRDLYHRVDV<br>IFCDKTIPNDPGFVVTLSNRMNYFQVAKTVAQRLNTDPMLLQFFKSQGYRDGPGNPLRHNYEGTLRDLLQFFK<br>PRQPKKLYYQQLKMKITDFENRRSFKCIWLNSQFREEEITLYPDKHGCVRDLLEECKKAVELGEKASGKLRLL<br>EIVSYKIIGVHQEDELLECLSPATSRTFRIEEIPLDQVDIDKENEMLVTVAHFHKEVFGTFGIPFLLRIHQGE<br>HFREVMKRIQSLLDIQEKEFEKFKFAIVMMGRHQYINEDEYEVNLKDFEPQPGNMSHPRPWLGLDHFNKAPKR<br>SRYTYLEKAIKIHN

Nucleotide sequences of the synthetic genes

ElaD (GenBank accession number: Q47013):

ATGGTGACCGTGGTGAGCAATTATTGTCAGCTGAGTCAGACCCAGCAGCTGAGTCAAACCTTCGCCGAAAAATTCACCGTGAC
CGAAGAACTGCTGCAGAGTCTGAAAAAAACCGCACTGAGCGGCGATGAAGAAAGCATTGAACTGCTGCATAATATTGCACTGG
GCTATGATAAATTCGGTAAAGAAGCCGAAGATATTCTGTATCATATTGTGCGTACCCCGACCAATGAAACCTTAAGCATTATT
CGCCTGATTAAAAATGCCTGCCTGAAACTGTATAATCTGGCCCATATTGCCACCAATAGCCCGCTGAAAAGCCATGATAGCGA
TGATCTGCTGTTCAAAAAACTGTTCAGCCCGAGTAAACTGATGACCATTATTGGTGATGAAATTCCGCTGATTAGCGAAAAAC
AGAGCCTGAGTAAAGTGCTGCTGAATGATGAAAATAATGAACTGAGTGATGGTACAAACTTCTGGGATAAAAATCGCCAGCTG
ACCACCGATGAAATTGCCTGTTATCTGCAGAAAATTGCCGCCAATGCAAAAAATACCCAGGTTAATTATCCGACCGGCCTGTA
TGTGCCGTATAGTACCCGTACCCATCTGGAAGATGCACTGAATGAAAATATTAAAAGCGATCCGAGCTGGCCGAATGAAGTTC
AGCTGTTCCCGATTAATACCGGTGGTCATTGGATTCTGGTGAGCCTGCAGAAAATCGTTAATAAAAAAAACAACAAGCTGCAG
ATCAAGTGTGTGATCTTCAATAGTCTGCGTGCCCTGGGTTATGATAAAGAAAATAGCCTGAAACGCGTGATTAATAGCTTCAA
TAGCGAACTGATGGGCGAAATGAGTAATAATAATATTAAGGTGCACCTGAACGAACCGGAAATTATCTTCCTGCACGCTGATC
TGCAGCAGTATCTGAGTCAGAGCTGTGGCGCCTTCGTGTGTATGGCCGCACAGGAAGTTATTGAACAGCGTGAAAGCAATAGT
GATAGCGCCCCGTATACCCTGCTGAAAAATCATGCAGATCGCTTCAAAAAATACAGTGCAGAAGAACAGTATGAAATTGACTT
CCAGCATCGCCTGGCAAATCGTAATTGCTATCTGGATAAATATGGCGATGCAAATATTAATCACTACTATCGCAATCTGGAAA
TTAAACATAGCCAGCCGAAAAATCGTGCAAGCGGCAAACGTGTGAGT

Lpg1148 (GenBank accession number: Q5ZWD7, 1-305):

ATGTATATGGATTTAGGGAGCGATAATATGGCATTAAGTAAAGAAATTCAGAATAAAAGTTTAAGATCATTAATCCAAAAATA
CGCCAGACAACTTAATGAAACAGACAAGAAGCTCATAGAACTTATTAATAATCTCGCTGCAGCAGATGATATTCCTTTGTATG
CCCAAAACCAAAATGTTATCAATTTGTTAATTAAAACCATTAAAGACAGCCAGTCTGAACACAATCCGGATTTGGAGGGAACT
TCCTATTTCATTTATGGATTGATCACTGCAGAAATGGAGGCTCAATCTGAAATAAACCGCCAAAGACATCTTATGCAGGGACG
TTTTGATGAAACAGCATTGCATTTAAGAACAGAGCGTGATCAAATGCAAGATCATGACATGACTCTGCTCATGCCAAAATCTC
AGGGGCGTATCGTTGTGATGGCAGTTCTCAATCGCTACGACTCTCATTCTGCTAATGCGATTATAGAAACTTTGGCAAGTGAT
GTATTCAATCCTGAAGTTCATTACATTATGATACCTGTTGGACCCGGACATTGGCGAGGAGTTTATTTAAGCAAACCCCAAGG
CGGCACTAGCGATACAGCTTATGATCTTGAATTATTTGACCCTTATGGGCCTGAAGGCGCTGCAGTACTTGATGACTATGTAC
TTGATTTATTAAACCAGTGTGGCGTTCCCAAAGAGCTTGTGAATATACGGCATACGGGTCCGAAACATCCGCAAGGTGATGCT
TACTCATGTGGGGATTTTACTTGTGCCTATAGCCATAAGAAAATGAAAGAATTTGGAGCTCCAGAAGGTAGTTATAATCCGAT
TCTTATCGATACGCTGGATAATTTAGGTAATGAAGATAATGTGCTTCGTATGACAACGCGCGAAGAAACGCGAGCGCTTGTTG
ATTAA

Lpg1621/Ceg23 (GenBank accession number: Q5ZV21, 1-368):

ATGCACACAAAAAAGGATAAAAAAGTAATATCACTTCAAGAAAGAGTAGAAAATGCAGTTGATGTTTCGGGGGCTTTTGATAA
CTGCTTTTTCCATAATTTCGCACTTTACCTGTTAACGAATAATTTACCACTTCCTGATGATTTATTTCACTTCAAATCAATCA
TTAATAGAAATAGTAAGGCAGAACAATTATTTGAATTTTTTCATAACCCGGAATCATTGAATTTATTTTCTATATTAGATAAG
GAAAATGATGTTTCTGAACCCTCTGGTTATCTCTTTGAAAAGAGCTTGATTTTAGGATTTCTGCTTAGGGAATGGTTCCCAAC
TCAACTTGTTAATAATTCAGCAGTTAAAGCGGAAATGCTGGAAGGTGAGAAAGGGGTATTCAGTGCTTTTAAAAATTATAAAG
AGTATCGCTCATTTATGTCGAAGGAAGAATTAAAATCAACGGAATTTGGTGCGTTATATGAAGCGAACGAGGCTTTTTTAGAG
TATTTTTATAATCGCTCTGAAAGTACTTTAATAAATAAGGATTCACCTTTTGAAAAATATTTTGTTGGCTCGTCCTCAGATGA
AGAGGCAATTAAAAATTATTGGGACGCAGAAGGGTATACATTATATTGTCAACATTTGGCGAAACCTCAGGTTAAATTATCTT
ATATTGAAATTATGACCATGATGAAGGTCATCAATCAGCCACTTACAATATATGATCGTTCTACTTCCTCAATTGTAGCCGAG
TATGTCAACCCAAAGGTCAATTTACCTGATTTTGAAGTGGCTATTGATGCTCTTCAAGGGCATTATTTTCTTTTAAAAACAGA
GGAAACGGAAAAAGAGTTAGAAGAATATGAAAGAAGTTATGCTCAATATAAAAGAGATAGAAGTGAAATCCTTGCACACTCCG
ATAAACCTGTTTCTTCCTTATTGGTTCGAGCTACTTGCCCCAAAGGTCATTTGGATGAAGATCCTTTTATTGCATTAATTGAA
AGCCTTTCCGAAATAAACAGCCTTAGCCAAATTGATACTAATTTGAAAAACGAGAATACAGATATTCCCAATTGCAACTTCCT
TTTAAAGGTAGGTGCTTCTGTAGTTTAA

RavD (GenBank accession number: Q5ZZ51):

ATGAACTTAAAAAAAGAAGTGTTCTTAAACCAAAATTGTGCAGAAATGATGATAAAGAAAGCTGCTCGGTTGATTTTAGGCAG
TGATCTTGATTTTGAATACACTCGAGATGTTCAAGATATTCAAATTGATCTGGGCCCTGCTTTTATGTTCAGTCCAGACGAAG
AAAAAACACTGTGGGTTAGTGGGAAAAATCAGGAAACGTTGGAAAGGGATCTGGCGACTCTTAATAAAAGCAGTGCCTACTTT
TTCCGGACTGGTACTCAAGGAGGGGCTGGTCATTGGCAAGTCCTGTACTATGAGGCTGCTAAATCAGGTTGGGTTAGCTATTC
ATCTCAATCCAATCACTTTCAAGTGACTGACAGCAATGGGAAGTTAACAGCCAGTGGTAAGGGACTGCTTGTTCCCCATGCCA
ATTGGGGAAAGGAAAATGGAAATTATGCATTTTTGCTCGTAAACGCAAGTGCACAAAACATCATTCATGCAGCGAATTTCGTG
TATATTTTCAGAACTCAGAATGAGGATGCTGCAATACAATATTGTGCGCTCAATCAGGCGCTTCATCCAGAAATAAAGAGAAT
AACTATAACCAAAGTTCAAACACAAACGAGGTTAACACCAGAGATTCATTGTCCCCGGGGAGATTTATCTTATGCGAATCTTA
AAAATGTATACAGTAAGCATCTAGCCTTGTTGCATGTTATCAACCGCCTGGAAGCAGGAAGTAAAAGCTACAATCCTTACTGG
ATGGGTAGTCAAGAGAAGCTCAATAAAATTTTTGAGGCACTTGGACATTGTTATCAAAAAGGCCTTGATGTCGATAATGAATT
AAACAATAGCAACAGCAAATTATCCAAAGCAATCAATATGCGCCGCCTCCCATCGCTTTTTAGTTTTTGGTGTGAAAAAAACG
AATCTTTCGTGGAATCAAGAAAAATAATTGAAGAACACAGGATTTGCAGGCTAAATAATTTATAA

RickCE (GenBank accession number: Q1RK35):

ATGGCACATCACCACCACCATCACGTGGGTACCGAAATCAAGGATTACTGGTATACCGAAAATGAAATTACCCATCTGCTGAC
CGCACAGCTGGATGAAAAAAAATTTAGCGTTCAGCCGGCCATTACCTTTCGTAATACCGCCCTGACCGAAGAAATGCTGAAAG
ATTATACCGCAAAAGGTGAAGAAAAAAATAAGATTCTGGCCGAAGTTCAGGAAACCATTAAAATTGCCAATCTGATTCCGGAT
AAAGAAGAACGCGCACTGATGCTGGGTGATGCCAAAAAACGTGAAGAAATTCTGAAACTGAGCGATGCAGAACGCGAAAAACT
GAAAAATGATCTGCTGCGCGGTGGCGAAGCACAGCAGCAGATTAATGAAGATATTCTGAATCGCGCAACCAAAGATATTAAAG
ATAATGGCAAAGAGGCCGCCGTGATTCCGATTGAAATGGGTTATGGCCATTGGACCGTGCTGGTTGCAAAATATGATAAAAAA
GATAACCAGATCATCCTGACCTTTAATGATAGCCTGGGCAATAGCATTAATTATGATGGCCAGAAACTGCCGAAACTGATTGA
TAAAACCCTGGGTAATCTGCCGAATAAACCGATTATTATTGATGAACAGACCAAACAGCAGACCGATCAGAGCGCATGTGGTG
TTTTTACCGTGGATAATGGTATTAAAATCGCAAAAGGTCAGGCCATTCTGAGTACCGAAGAAAGCAAAGGTGAAAAAGGTCTG
CGCCTGCGCGAACATCATGCCCAGATTCTGACCGATGCCATGTTTAAACAGGATGCCCAGTGGATTCGTCAGCAG

RickULP (GenBank accession number: ABV76659.1):

ATGGCACATCACCACCACCATCACGTGGGTACCCAGCAGCAGGCACCTGCAAATAATCAGAAACCGTGGGAAAAACTGGGTAT
TCCGCAGGAAATGTATAAAGAAAGCCTGAAAGCAGAACAGCAGCTGGCAAAACCGATTATTGAACCGAAACAGCAGATTCCGG
AAAAAAAAAGTAGTCTGGTTATTAATACCGAGGATCAGGTTGGCGTGTATAATACCGGTAATATTAAACAGCCGACCTATCTG
TATACCGAAGATGATATTAAAAACATCCTGGAAGCAAATATCGATAAAAATATGTTCAGCATCTTCCATCATGCCAGTCTGGA
AGAACCGGAAATTCTGAAAGATACCCTGCGCGTGACCGTGGAAGATCTGATTCTGGATAATAAACCGGCCATTATTCCGCTGA
ATACCGGCCATAAACATTGGCTGCTGCTGATGGCAAGCAAAGATGATAAAGGTAATATTAACTTCATGTACAACGACCCGTAT
GGTGAACCGCTGGAAAGTCAGCCGAAAGTTACCGAATATATTACCGAAATTTACCCGGATGCCAAAATTACCGATCTGAATAC
CAAACAGCAGGCCAATGTTTATGATTGCGGCGTTTTTGTTTGCGATAGCGCAATTAAACTGAGTAAAGGTCAGAAAATTCTGA
CCACCGAAGAAAGCAAAGATCAGGGTATTAATCTGCGCCAGGCCCAGGCCAATACCCTGCTGATTCAGCAGCAGGCGATTACC
ATTGGTCATGAA

SENP1 (GenBank accession number: Q9P0U3):

ATGTCGTACTACCATCACCATCACCATCACGATTACGATATCCCAACGACCGAAAACCTGTATTTTCAGGGCGCCATGGCTGA
TATCGGATCCGATAGTGAAGATGAATTTCCTGAAATTACAGAGGAAATGGAGAAAGAAATAAAGAATGTATTTCGTAATGGGA
ATCAGGATGAAGTTCTCAGTGAAGCATTTCGCCTGACCATTACACGCAAAGATATTCAAACTCTAAACCATCTGAATTGGCTC
AATGATGAGATCATCAATTTCTACATGAATATGCTGATGGAGCGAAGTAAAGAGAAGGGCTTGCCAAGTGTGCATGCATTTAA
TACCTTTTTCTTCACTAAATTAAAAACGGCTGGTTATCAGGCAGTGAAACGTTGGACAAAGAAAGTAGATGTATTTTCTGTTG
ACATTCTTTTGGTGCCCATTCACCTGGGAGTACACTGGTGTCTAGCTGTTGTGGACTTTAGAAAGAAGAATATTACCTATTAC
GACTCCATGGGTGGATAAACAATGAAGCCTGCAGAATACTCTTGCAATACCTAAAGCAAGAAAGCATTGACAAGAAAAGGAA
AGAGTTTGACACCAATGGCTGGCAGCTTTTCAGCAAGAAAAGCCAGGAGATTCCTCAGCAGATGAATGGAAGTGACTGTGGGA
TGTTTGCCTGCAAATATGCTGACTGTATTACCAAAGACAGACCAATCAACTTCACACAGCAACACATGCCATACTTCCGGAAG
CGGATGGTCTGGGAGATCCTCCACCGAAAACTCTTG

SnCE1 (GenBank accession number: F8L4W9):

GGGCCCCTGGGATCCAGCCTGACCAGCAAAGCAATTTGCCGCTATAGCCTGAGCAGCGTGGAAAGTATTCAGCCGAAAACCCA
GGTTAAAGCCATTGAACCGAAAGGTGAAAAAGATCGTTTTCTGATTGATGAACGTAATTGGCTGAATAATCAGCATCTGGGCC
TGTATAGTCTGTTTCTGCAGGAAAAATATGGCCCGGAAGTTTTCTTTCCGTTTGGCGGTTGGACCTATGTTTTTCCGGGTCTG
ACCGATCGCTTTTTCAAAGAAGATAGCTATCATATTCTGGATGTTCGCGCAAAACGTATTAAGAGTTTTCTGGATTATAAGAG
TATCACCTATCCGCTGTTTATTGGTGGCAATCATTGGGGCCTGCTGTTTATTGATCGCGAAAAACGCACCGTTGAATATTATG
ATAGTAAAATTAACTACGGTAACTACGAAGAAGGCCTGCAGGGTATTAAGGATGTGGCCGCAAAATTCACTAAATATGATCCG
GGCGAAAAACCGTATACCTATCTGGAAAAAATTAAGAAAAAGCTGCAGCCGGATGGTTATCAGTGCGGTCCGTGGGCACTGTA
TTTTCTGGAACATCGCCTGGAAAATCCGGAAGTTGATTTTAATCAGCTGGATCTGAATGAAGCACAGAATATGATTGCAAAAT
ACCGTTTTGCCGTTCGCGATAAACTGCTGGAACTGCAGAAAAATGGCAATACCCTGTATTGT

SplA-SUMO (GenBank accession number: WP_399659753.1, N-terminal SUMO-tag):

ATGGGTCATCATCATCATCATCACGGCAGCGGCCTGGTGCCGCGCGGCAGCGCTAGCATGTCGGACTCAGAAGTCAATCAAGA
AGCTAAGCCAGAGGTCAAGCCAGAAGTCAAGCCTGAGACTCACATCAATTTAAAGGTGTCCGATGGATCTTCAGAGATCTTCT
TCAAGATCAAAAAGACCACTCCTTTAAGAAGGCTGATGGAAGCGTTCGCTAAAAGACAGGGTAAGGAAATGGACTCCTTAAGA
TTCTTGTACGACGGTATTAGAATTCAAGCTGATCAGACCCCTGAAGATTTGGACATGGAGGATAACGATATTATTGAGGCTCA
CAGAGAACAGATTGGTGGTGAAAAGAACGTTAAGGAAATCACCGATGCAACCAAAGAACCGTATAATAGCGTGGTTGCCTTCG
TTGGCGGCACCGGTGTTGTGGTGGGCAAAAATACCATTGTTACCAATAAACATATCGCAAAAAGCAATGATATCTTCAAAAAC
CGTGTGAGCGCCCATCATAGCAGTAAAGGCAAAGGCGGTGGTAATTATGATGTGAAAGATATTGTTGAGTACCCGGGTAAAGA
AGATCTGGCAATTGTTCATGTTCATGAAACCAGCACCGAAGGTCTGAACTTCAATAAAAATGTTAGCTATACCAAGTTCGCCG
ATGGTGCAAAAGTTAAAGATCGTATTAGTGTGATTGGTTATCCGAAAGGTGCCCAGACCAAATATAAAATGTTCGAAAGTACC
GGTACCATTAATCATATTAGTGGCACCTTCATGGAATTCGATGCCTATGCCCAGCCGGGCAATAGTGGCAGCCCGGTGCTGAA
TAGCAAACATGAACTGATTGGCATTCTGTATGCCGGTAGCGGTAAAGATGAAAGCGAAAAAAACTTCGGCGTGTACTTCACCC
CGCAGCTGAAAGAATTCATTCAGAATAATATCGAGAAGGGCAGTTGGAGCCATCCGCAGTTTGAAAAAGGCGGCGGCAGTGGC
GGCGGCAGTGGCGGCAGTAGCGCCTGGAGCCATCCGCAGTTTGAAAAA

SplB-SUMO (GenBank accession number: WP_271285242.1, N-terminal SUMO-tag):

ATGGGTCATCATCATCATCATCACGGCAGCGGCCTGGTGCCGCGCGGCAGCGCTAGCATGTCGGACTCAGAAGTCAATCAAGA
AGCTAAGCCAGAGGTCAAGCCAGAAGTCAAGCCTGAGACTCACATCAATTTAAAGGTGTCCGATGGATCTTCAGAGATCTTCT
TCAAGATCAAAAAGACCACTCCTTTAAGAAGGCTGATGGAAGCGTTCGCTAAAAGACAGGGTAAGGAAATGGACTCCTTAAGA
TTCTTGTACGACGGTATTAGAATTCAAGCTGATCAGACCCCTGAAGATTTGGACATGGAGGATAACGATATTATTGAGGCTCA
CAGAGAACAGATTGGTGGTGAAAATAACGTTACCAAGGTGAAAGATACCAATATCTTCCCGTATACCGGTGTTGTTGCCTTCA
AAAGCGCCACCGGCTTCGTTGTGGGTAAAAATACCATTCTGACAAATAAACACGTGAGCAAAAATTATAAGGTGGGTGATCGT
ATTACCGCCCATCCGAATAGCGATAAAGGCAATGGTGGTATCTATAGTATTAAAAAAATCATCAACTACCCTGGTAAGGAAGA
TGTTAGTGTTATTCAGGTGGAAGAACGTGCAATTGAACGCGGCCCGAAAGGCTTCAACTTCAATGATAATGTTACCCCGTTCA
AATATGCAGCCGGCGCCAAAGCCGGCGAACGTATTAAAGTTATTGGCTATCCGCATCCGTATAAAAATAAATATGTGCTGTAC
GAAAGCACCGGTCCGGTGATGAGCGTTGAAGGTAGCAGTATTGTGTATAGTGCACATACCGAAAGTGGCAATAGTGGTAGTCC
GGTTCTGAATAGCAATAATGAACTGGTTGGCATTCACTTCGCAAGCGATGTGAAAAATGATGATAATCGCAATGCATATGGTG
TGTACTTCACCCCGGAAATTAAAAAATTCATTGCCGAAAATATCGACAAGGGCAGTTGGAGCCATCCGCAGTTTGAAAAAGGC
GGCGGCAGTGGCGGCGGCAGTGGCGGCAGTAGCGCCTGGAGCCATCCGCAGTTTGAAAAA

SseL (GenBank accession number: Q8ZNG2):

ATGGCACATCACCACCACCATCACGTGGGTACCATGAGCGATGAAGCACTGACCCTGCTGTTTAGTGCCGTTGAAAATGGTGA
CCAGAATTGTATTGATCTGCTGTGTAATCTGGCCCTGCGTAATGATGATCTGGGCCATCGTGTTGAAAAATTTCTGTTTGATC
TGTTTAGCGGTAAACGCACCGGTAGCAGCGATATTGATAAAAAGATTAATCAGGCCTGTCTGGTGCTGCATCAGATTGCCAAT
AATGATATTACCAAAGATAACACCGAGTGGAAAAAACTGCACGCTCCGAGTCGCCTGCTGTATATGGCAGGCAGCGCCACCAC
CGATCTGAGTAAAAAGATTGGTATTGCCCATAAAATCATGGGCGATCAGTTTGCACAGACCGATCAGGAACAGGTGGGTGTTG
AAAATCTGTGGTGTGGCGCCCGTATGCTGAGTAGCGATGAACTGGCCGCCGCCACCCAGGGTCTGGTTCAGGAAAGCCCGCTG
CTGAGCGTTAATTATCCGATTGGTCTGATTCATCCGACCACCAAAGAAAATATTCTGAGTACCCAGCTGCTGGAAAAAATTGC
ACAGAGTGGTCTGAGTCATAATGAAGTTTTTCTGGTTAATACCGGCGATCATTGGCTGCTGTGCCTGTTTTATAAACTGGCCG
AAAAAATTAAGTGCCTGATTTTTAATACCTACTACGATCTGAATGAGAATACCAAACAGGAAATTATTGAGGCAGCAAAAATT
GCAGGCATTAGCGAAAGCGATGAAGTGAATTTTATTGAAATGAACCTGCAGAATAACGTTCCGAATGGTTGTGGTCTGTTTTG
CTATCATACCATTCAGCTGCTGAGCAATGCCGGTCAGAATGATCCGGCAACCACCCTGCGTGAATTTGCAGAAAATTTTCTGA
CCCTGAGTGTTGAAGAACAGGCACTGTTTAATACCCAGACCCGTCGTCAGATCTATGAATATAGTCTGCAG

USP48 (GenBank accession number: PQ658229):

ATGGCCCCGCGGCTGCAGCTGGAGAAGGCGGCCTGGCGCTGGGCGGAGACGGTGCGGCCCGAGGAGGTGTCGCAGGAGCACAT
CGAGACCGCTTACCGCATCTGGCTGGAGCCCTGCATTCGCGGCGTGTGCAGACGAAACTGCAAAGGAAATCCGAATTGCTTGG
TTGGTATTGGTGAGCATATTTGGTTAGGAGAAATAGATGAAAATAGTTTTCATAACATCGATGATCCCAACTGTGAGAGGAGA
AAAAAGAACTCATTTGTGGGCCTGACTAACCTTGGAGCCACTTGTTATGTCAACACATTTCTTCAAGTGTGGTTTCTCAACTT
GGAGCTTCGGCAGGCACTCTACTTATGTCCAAGCACTTGTAGTGACTACATGCTGGGAGACGGCATCCAAGAAGAAAAAGATT
ATGAGCCTCAAACAATTTGTGAGCATCTCCAGTACTTGTTTGCCTTGTTGCAAAACAGTAATAGGCGATACATTGATCCATCA
GGATTTGTTAAAGCCTTGGGCCTGGACACTGGACAACAGCAGGATGCTCAAGAATTTTCAAAGCTCTTTATGTCTCTATTGGA
AGATACTTTGTCTAAACAAAAGAATCCAGATGTGCGCAATATTGTTCAACAGCAGTTCTGTGGAGAATATGCCTATGTAACTG
TTTGCAACCAGTGTGGCAGAGAGTCTAAGCTTTTGTCAAAATTTTATGAGCTGGAGTTAAATATCCAAGGCCACAAACAGTTA
ACAGATTGTATCTCGGAATTTTTGAAGGAAGAAAAATTAGAAGGAGACAATCGCTATTTTTGCGAGAACTGTCAAAGCAAACA
GAATGCAACAAGAAAGATTCGACTTCTTAGCCTTCCTTGCACTCTGAACTTGCAGCTAATGCGTTTTGTCTTTGACAGGCAAA
CTGGACATAAGAAAAAGCTGAATACCTACATTGGCTTCTCAGAAATTTTGGATATGGAGCCTTATGTGGAACATAAAGGTGGG
TCCTACGTGTATGAACTCAGCGCAGTCCTCATACACAGAGGAGTGAGTGCTTATTCTGGCCACTACATCGCCCACGTGAAAGA
TCCACAGTCTGGTGAATGGTATAAGTTTAATGATGAAGACATAGAAAAGATGGAGGGGAAGAAATTACAACTAGGGATTGAGG
AAGATCTAGCAGAACCTTCTAAGTCTCAGACACGTAAACCCAAGTGTGGCAAAGGAACTCATTGCTCTCGAAATGCATATATG
TTGGTTTATAGACTGCAAACTCAAGAAAAGCCCAACACTACTGTTCAAGTTCCAGCCTTTCTTCAAGAGCTGGTAGATCGGGA
TAATTCCAAATTTGAGGAGTGGTGTATTGAAATGGCTGAGATGCGTAAGCAAAGTGTGGATAAAGGAAAAGCAAAACACGAAG
AGGTTAAGGAGCTGTACCAAAGGTTACCTGCTGGAGCTGAGCCCTATGAGTTTGTCTCTCTGGAATGGCTGCAAAAGTGGTTG
GATGAATCAACACCTACCAAACCTATTGATAATCACGCTTGCCTGTGTTCCCATGACAAGCTTCACCCGGATAAAATATCAAT
TATGAAGAGGATATCTGAATATGCAGCTGACATTTTCTATAGTAGATATGGAGGAGGTCCAAGACTAACTGTGAAAGCCCTGT
GTAAGGAATGTGTAGTAGAACGTTGTCGCATATTGCGTCTGAAGAACCAACTAAATGAAGATTATAAAACTGTTAATAATCTG
CTGAAAGCAGCAGTAAAGGGCAGCGATGGATTTTGGGTGGGGAAGTCCTCCTTGCGGAGTTGGCGCCAGCTAGCTCTTGAACA
GCTGGATGAGCAAGATGGTGATGCAGAACAAAGCAACGGAAAGATGAACGGTAGCACCTTAAATAAAGATGAATCAAAGGAAG
AAAGAAAAGAAGAGGAGGAATTAAATTTTAATGAAGATATTCTGTGTCCACATGGTGAGTTATGCATATCTGAAAATGAAAGA
AGGCTTGTTTCTAAAGAGGCTTGGAGCAAACTGCAGCAGTACTTTCCAAAGGCTCCTGAGTTTCCAAGTTACAAAGAGTGCTG
TTCACAGTGCAAGATTTTAGAAAGAGAAGGGGAAGAAAATGAAGCCTTACATAAGATGATTGCAAACGAGCAAAAGACTTCTC
TCCCAAATTTGTTCCAGGATAAAAACAGACCGTGTCTCAGTAACTGGCCAGAGGATACGGATGTCCTCTACATCGTGTCTCAG
TTCTTTGTAGAAGAGTGGCGGAAATTTGTTAGAAAGCCTACAAGATGCAGCCCTGTGTCATCAGTTGGGAACAGTGCTCTTTT
GTGTCCCCACGGGGGCCTCATGTTTACATTTGCTTCCATGACCAAAGAAGATTCTAAACTTATAGCTCTCATATGGCCCAGTG
AGTGGCAAATGATACAAAAGCTCTTTGTTGTGGATCATGTAATTAAAATCACGAGAATTGAAGTGGGAGATGTAAACCCTTCA
GAAACACAGTATATTTCTGAGCCCAAACTCTGTCCAGAATGCAGAGAAGGCTTATTGTGTCAGCAGCAGAGGGACCTGCGTGA
ATACACTCAAGCCACCATCTATGTCCATAAAGTTGTGGATAATAAAAAGGTGATGAAGGATTCGGCTCCGGAACTGAATGTGA
GTAGTTCTGAAACAGAGGAGGACAAGGAAGAAGCTAAACCAGATGGAGAAAAAGATCCAGATTTTAATCAAAGCAATGGTGGA
ACAAAGCGGCAAAAGATATCCCATCAAAATTATATAGCCTATCAAAAGCAAGTTATTCGCCGAAGTATGCGACATAGAAAAGT
TCGTGGTGAGAAAGCACTTCTCGTTTCTGCTAATCAGACGTTAAAAGAATTGAAAATTCAGATCATGCATGCATTTTCAGTTG
CTCCTTTTGACCAGAATTTGTCAATTGATGGAAAGATTTTAAGTGATGACTGTGCCACCCTAGGCACCCTTGGCGTCATTCCT

Topological maps of the target proteins

Topological maps of the four candidate proteins that were used to determine Spl cleavage sites (Table 2) were generated using ESPript 3.0 (Robert and Gouet 2014). The putative cleavage sites (identified by MS) and confirmed cleavage sites (by site-directed mutagenesis) for SplA and SplB are marked with arrows and the type of Spl (A or B).

RickULP cleavage by Spls

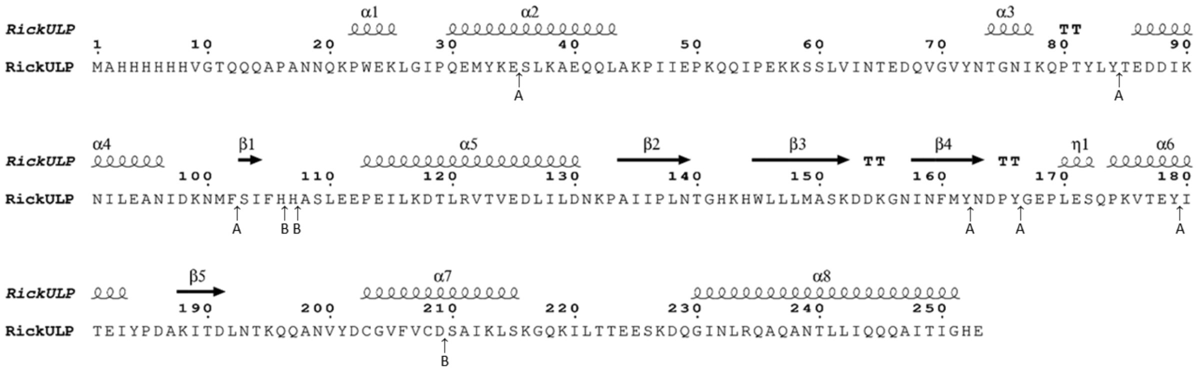

RickCE cleavage by Spls:

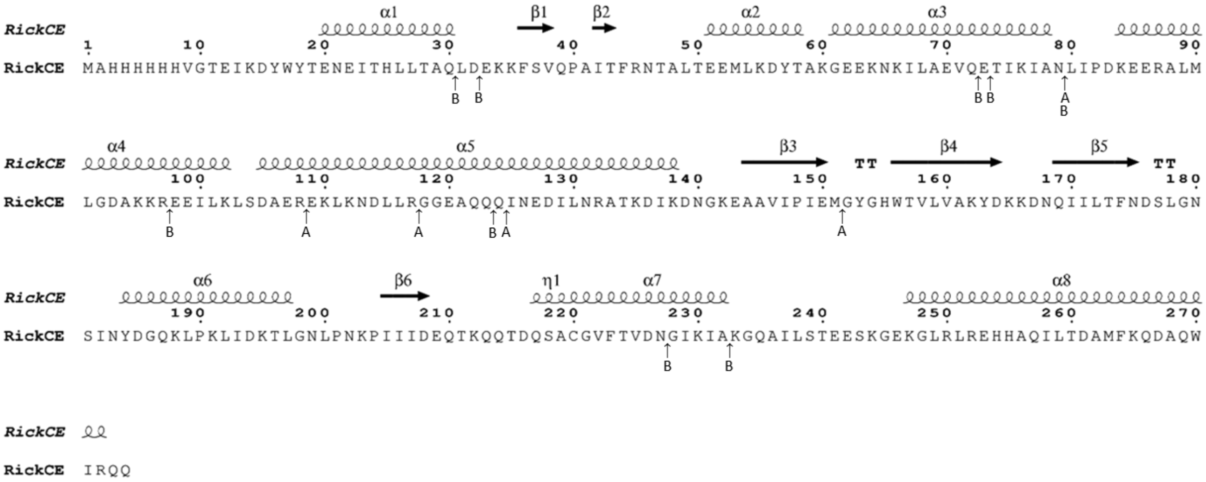

SseL cleavage by Spls:

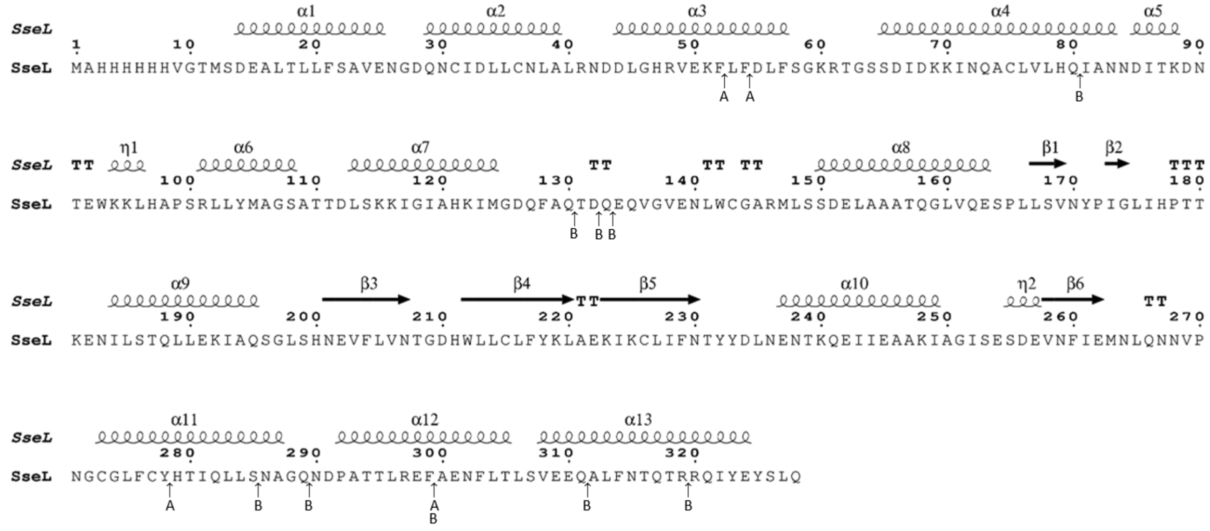

SENP1 cleavage by Spls:

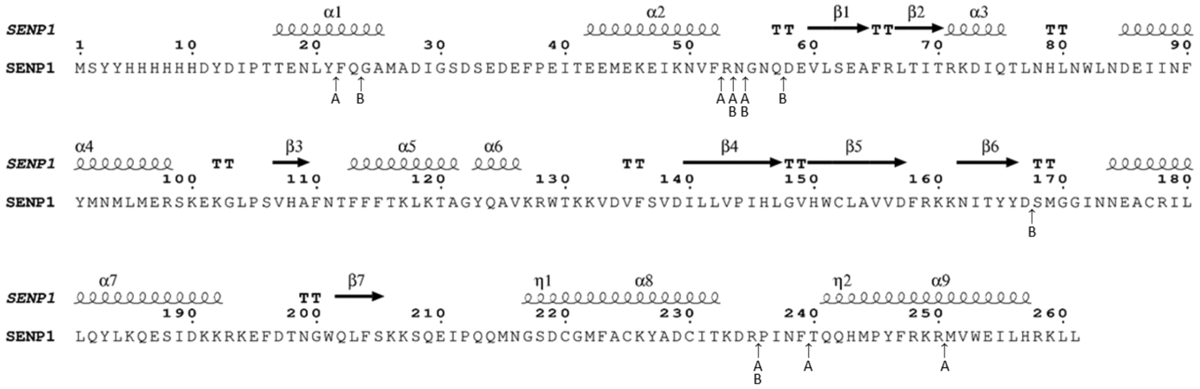

Instrument settings for mass spectrometry analysis

Table S2 Reverse phase liquid chromatography (RPLC) settings.

Instrument	Ultimate 3000 RSLC (Thermo Scientific)	
Trap column	75 µm inner diameter, packed with 3 µm C18 particles (Acclaim PepMap100, Thermo Scientific)	
Analytical column	Accucore 150-C18, (Thermo Fisher Scientific)<br>25 cm x 75 µm, 2,6 µm C18 particles, 150 Å pore size	
Buffer system	binary buffer system consisting of 0.1% acetic acid in HPLC-grade water (buffer A) and 100% ACN in 0.1% acetic acid (buffer B)	
Flow rate	300 nl/min	
Gradient	HUNTER samples<br>0 min 2% B →<br>2 min 5% B →<br>10 min 7% B →<br>40 min 25% B →<br>45 min 40% B →<br>47 min 90% B →<br>53 min 90% B →<br>55 min 2% B →<br>65 min 2% B	gel slices<br>0 min 2% B →<br>2 min 5% B →<br>10 min 5% B →<br>70 min 25% B →<br>75 min 40% B →<br>77 min 90% B →<br>82 min 90% B →<br>83 min 2% B →<br>90 min 2% B
Gradient duration	65 min	90 min
Column oven temperature	40°C	

Table S3 Mass spectrometry settings.

	HUNTER samples	gel slices
Instrument	Exploris 480 mass spectrometer (Thermo Scientific)	Q Exactive Plus mass spectrometer (Thermo Scientific)
Electrospray	Nanospray Flex Ion Source	
Operation mode	Data dependent acquisition	
Method duration	60 min	90 min
Full MS		
MS scan resolution	120000	70,000
AGC target	300 %	3e6
RF Lens	40 %	
maximum ion injection time for the MS scan	auto	120 ms
Scan range	350 to 1200 m/z	300 to 1650 m/z
Spectra data type	profile	
dd-MS2		
Number of dependent scans	15	10
Resolution	15,000	17,500
Maximum ion injection time mode	auto	120 ms
Scan range	350 to 1200 m/z	200 to 2000 m/z
Data type	centroid	
Time	22 ms	none
Microscans	1	
Isolation window	1.4 m/z overlap	3 m/z
First mass	110 m/z	100 m/z
Dissociation mode	higher energy collisional dissociation (HCD)	
HCD normalized collision energy	30	27,5
Dynamic exclusion	10 s	30 s
Apex trigger	none	1 to 40 s
Charge state	include 2-6	
Intensity threshold	5000	none
Multiple charge states	None	all
Peptide Match	None	preferred

References

Dubin G, Stec-Niemczyk J, Kisielewska M, Pustelny K, Popowicz GM, Bista M, Kantyka T, Boulware KT, Stennicke HR, Czarna A, Phopaisarn M, Daugherty PS, Thøgersen IB, Enghild JJ, Thornberry N, Dubin A, Potempa J (2008) Enzymatic activity of the *Staphylococcus aureus* SplB serine protease is induced by substrates containing the sequence Trp-Glu-Leu-Gln. J Mol Biol 379:343–356. doi: 10.1016/j.jmb.2008.03.059

Robert X, Gouet P (2014) Deciphering key features in protein structures with the new ENDscript server. Nucleic Acids Res 42:W320-4. doi: 10.1093/nar/gku316

Schechter I, Berger A (1967) On the size of the active site in proteases. I. Papain. Biochem Biophys Res Commun 27:157–162. doi: 10.1016/S0006-291X(67)80055-X
